# Applications of Phenomenological Loudness Models to Cochlear Implants

**DOI:** 10.3389/fpsyg.2020.611517

**Published:** 2021-01-13

**Authors:** Colette M. McKay

**Affiliations:** ^1^Bionics Institute, Melbourne, VIC, Australia; ^2^Department of Medical Bionics, University of Melbourne, Melbourne, VIC, Australia

**Keywords:** Cochlear implants, loudness, intensity, temporal resolution, models

## Abstract

Cochlear implants electrically stimulate surviving auditory neurons in the cochlea to provide severely or profoundly deaf people with access to hearing. Signal processing strategies derive frequency-specific information from the acoustic signal and code amplitude changes in frequency bands onto amplitude changes of current pulses emitted by the tonotopically arranged intracochlear electrodes. This article first describes how parameters of the electrical stimulation influence the loudness evoked and then summarizes two different phenomenological models developed by McKay and colleagues that have been used to explain psychophysical effects of stimulus parameters on loudness, detection, and modulation detection. The Temporal Model is applied to single-electrode stimuli and integrates cochlear neural excitation using a central temporal integration window analogous to that used in models of normal hearing. Perceptual decisions are made using decision criteria applied to the output of the integrator. By fitting the model parameters to a variety of psychophysical data, inferences can be made about how electrical stimulus parameters influence neural excitation in the cochlea. The Detailed Model is applied to multi-electrode stimuli, and includes effects of electrode interaction at a cochlear level and a transform between integrated excitation and specific loudness. The Practical Method of loudness estimation is a simplification of the Detailed Model and can be used to estimate the relative loudness of any multi-electrode pulsatile stimuli without the need to model excitation at the cochlear level. Clinical applications of these models to novel sound processing strategies are described.

## Introduction

Cochlear implants (CIs) have been one of the most successful medical devices developed over the last 40 years, now approaching a million users worldwide. CIs restore hearing sensation to severely or profoundly deaf people by electrically stimulating residual hearing nerves in the cochlea. Although there are many variations of signal processing strategies, which encode features of sounds into patterns of electrical stimulation, all are based upon a simple principle: amplitude variations in different acoustic frequency bands are encoded as current amplitude variations of electrical pulse trains (or rarely sinusoids) on tonotopically assigned intracochlear electrodes. Thus, in addition to the tonotopic assignment of frequency bands to intra-cochlear electrode position, intensity coding is the main means of transferring acoustic stimulus feature information to the electrical stimulus and hence to the perception of the CI user. This article summarizes features of intensity and loudness coding in CIs and places this knowledge in the context of two phenomenological loudness models developed and validated by McKay and collaborators. These models throw light on how the perception of loudness and temporal information are modulated by parameters of electrical stimulation and how the neural processing of sounds differs from that for acoustic stimulation. It should be noted that the psychophysical perception of loudness can vary with the context in which a sound is heard (Schneider and Parker, 1990; Wang and Oxenham, 2016) and with slow acting changes in central gain (Pieper et al., 2018; Auerbach et al., 2019). However, this review focuses on the influence of electrical stimulus parameters on perceived loudness and on the transmission of temporal features in sounds.

## Single-Electrode Stimuli

### Loudness of Simple Single-Electrode Stimuli

**FIGURE 1:**
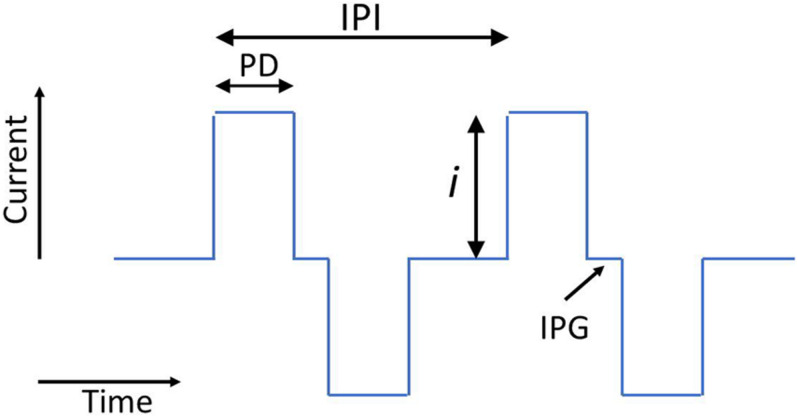
Schematic showing two biphasic current pulses and the parameters current (i), pulse duration (PD), interphase gap (IPG), and interpulse interval (IPI).

The electrical stimuli in the majority of commercial CI systems are composed of cathodic-first biphasic pulse trains. The biphasic pulses are defined by pulse duration (PD), current amplitude (i), interphase gap (IPG) (Figure 1), and the mode of stimulation. The mode defines the current return path from the activated intracochlear electrode: monopolar (MP) mode (the most common) uses a return electrode, or electrodes, situated outside the cochlea; bipolar (BP) mode uses a nearby intracochlear electrode; and multipolar modes use a combination of return-path and/or active electrodes. The mode of stimulation controls the spatial specificity of the current path. To complete the description of a pulse train on a single active electrode, the interpulse intervals (IPIs) are required. All of these five parameters (i, PD, IPG, mode, and IPI) influence the loudness evoked by the stimulus. Although commercial systems generally use cathodic-first biphasic pulses in MP or BP modes, researchers have evaluated the effect on neural excitation of alternative pulse shapes and multipolar modes (e.g., Bonnet et al., 2004; Macherey et al., 2010; Srinivasan et al., 2010; Undurraga et al., 2012; Fielden et al., 2013; Marozeau et al., 2015; Carlyon et al., 2017, 2018). Different pulse shapes and multipolar modes influence both the amount of excitation induced by a current pulse and the spatial specificity of the neural activation. In general, multipolar modes can improve the spatial specificity of activated neural populations, but at the expense of higher currents being required to achieve the same loudness (Srinivasan et al., 2010; Fielden et al., 2013; Marozeau et al., 2015). Anodic-first biphasic pulses, triphasic pulses, and pseudo-monophasic pulses have all been compared to biphasic pulses in studies that have shown that different pulse shapes can affect place specificity, the location of the peak excitation, and loudness (Macherey et al., 2010, 2011; Undurraga et al., 2012; Carlyon et al., 2017). However, these alternative pulse shapes and modes are not yet used in commercial systems, and this review will mostly not consider their effects in detail, except where specified.

In general sound processor usage, with few exceptions, the value of the current amplitude (i) is used to control the loudness evoked by the stimulus and to convey amplitude modulations of temporal envelopes within each frequency band, while other stimulus parameters are fixed (Wouters et al., 2015). Over the relatively small current range between hearing threshold and maximum loudness for a simple pulse train on a single electrode, the relation between current and loudness can be well described by either a power or exponential function (Kwon and van den Honert, 2006). However, as described and explained in more detail in section “Multi-Electrode Stimuli,” the relation is more complex over the wider range of current amplitudes that can be used in complex multi-electrode or high-rate stimuli, with a power function describing the relation for low currents and an expansive function needed at high levels (McKay et al., 2003).

Since electrical charge is the means by which neurons are activated, it could be expected that changes in PD would have the same effect on loudness as changes in current (since both are linearly related to the total charge delivered). However, longer pulses are less effective at activating neurons than shorter pulses of equal total charge (Pfingst et al., 1991; Moon et al., 1993). This reduction in efficiency is well explained by the neural “leaky integrator” model (Miller et al., 2001). The ability with which neurons integrate charge on their membranes depends on the site of activation (dendrite, cell body or axon) and physical attributes of the neurons such as size and health, for example presence or absence of myelin (Parkins and Colombo, 1987; Horne et al., 2016). These neural properties lead to the amount of PD change versus current change for equivalent loudness change being different at different absolute current amplitudes and PDs (McKay and McDermott, 1999; Carlyon et al., 2005), and between different people and different electrode positions in the same person (Schvartz-Leyzac and Pfingst, 2016). The dependence of the effect of changing PD on neural health status has led to several proposals to use this effect in psychophysical or electrophysiological measures to evaluate neural health in individual CI users (Moon et al., 1993; McKay and McDermott, 1998; Prado-Guitierrez et al., 2006; Ramekers et al., 2014). In a similar way, an increase of the IPG between the two phases of the biphasic pulse leads to more effective activation of neurons (McKay and Henshall, 2003; Carlyon et al., 2005), possibly because the second phase can remove charge from the neuron before it fires. The influence of the IPG has also been shown in animal studies to be correlated with neural health (Prado-Guitierrez et al., 2006; Ramekers et al., 2014; Schvartz-Leyzac and Pfingst, 2016; Hughes et al., 2018), and the effect has been proposed as a measure of neural health in humans, in a similar way to the PD effect (Hughes et al., 2018; He et al., 2020; Schvartz-Leyzac et al., 2020).

**FIGURE 2:**
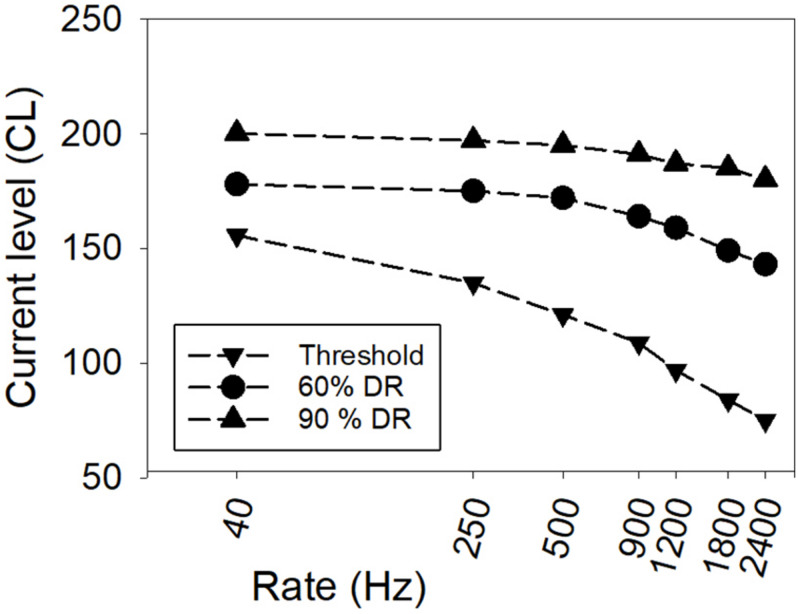
An example from one CI user showing threshold and equal-loudness functions versus rate of stimulation. Currents are depicted in clinical current level (CL) units, where one CL is 0.176 dB (Data from McKay et al., 2013a).

The rate of stimulation (controlled by the IPI) also affects the loudness evoked by a stimulus, with loudness increasing with increasing rate (Shannon, 1985). Figure 2 shows representative data for one CI user, illustrating how hearing threshold and equally loud currents typically change with rate of stimulation for biphasic pulse trains. Given that the phase duration and IPG are generally fixed for individuals in clinical use, the loudness of stimuli depends on the currents used, the time intervals between pulses, and the duration of the pulse train. The response state of auditory neurons (changing the probability of firing, and altering the total excitation elicited by an individual electrical pulse) depends on what has already occurred in the time leading up to a particular electrical pulse, with refractoriness reducing firing probability for neurons that have recently fired, facilitation increasing firing probability for very short IPIs, and adaptation lowering firing probability over sustained durations of stimulation (Tang et al., 2006; Boulet et al., 2016).

### The Temporal Model

A phenomenological model was developed by McKay and McDermott (1998) to explain the effect on loudness of IPIs in 2-pulse-per-period stimuli, and was later generalized by McKay et al. (2013b) to model the effects of rate of stimulation and stimulus duration on loudness or hearing threshold, effects of modulation frequency on modulation detection, and effects of masker stimulus features on forward masked thresholds. This model, designated here as the Temporal Model, describes how temporal factors in single-electrode stimuli influence psychophysical data. The model was based on similar acoustic models (Oxenham and Moore, 1994, 1995; Moore et al., 1996; Oxenham, 2001; Plack et al., 2002) in which the cochlear excitation evoked by a stimulus is integrated by a sliding temporal integration window and perceptual decisions (e.g., equal loudness, discrimination, and detection) are made by applying criteria to the output of the integrator. These authors showed that, if the integration occurred after the non-linear cochlear processes (instead of on the acoustic waveform), the integration window is invariant with acoustic level and frequency. Plack et al. (2002) argued that the linear integration window should act upon the intensity of basilar membrane vibration, which in turn may be linearly related to auditory nerve firing rate (Muller et al., 1991). Therefore, in the development of the Temporal Model applied to electric stimulation, the same central temporal integration window was applied to peripheral neural activity evoked by electrical current pulses, on the assumption that processing in the central auditory system is largely unaffected by peripheral hearing loss. Similar central decision criteria to those used in acoustic hearing could then be applied to the integrator output.

The integration window used in the Temporal Model has the following form:

(1) $$W\,\left(t\right)=\left(1-w\right)\times \exp\left(t/T_{b\,1}\right)+w\times e\,x\,p\,\left(t/T_{b\,2}\right),t<0W\,\left(t\right)=\text{exp}\,\left(-t/T_{a}\right),t\geq 0$$

where *T*_*a*_ and *T_*b*1_* together define the short time constant associated with temporal resolution, *T_*b*2_* defines a longer tail of the window associated with forward masking and the effect of stimulus duration, and *w* is the weighting of the long versus short time constants. For example, Oxenham (2001) derived the integration window shape to best fit forward masking data for normally hearing listeners: the best fitting values of the parameters were *T*_*a*_ = 3.5 ms, *T_*b*1_* = 4.6 ms, *T_*b*2_* = 16.6 ms, and *w* = 0.17.

To predict the effect of a stimulus parameter on detection, loudness, or discrimination using the Temporal Model the following four steps are used:

1. Using a reference stimulus, calculate the excitation evoked by each pulse relative to the first pulse. In practice this step involves modeling the peripheral effects of refractoriness, facilitation, adaptation, or amplitude modulation to describe how neural excitation changes with each pulse.
2. Integrate the excitation with the sliding temporal integration window in Eq. 1, the output of which is a function of integrated excitation versus time.
3. Apply the desired decision criterion to the integrator output. Such criteria will depend on the experiment being undertaken.
4. Repeat with different values of the stimulus parameter under investigation to achieve the aimed-for criterion at the integrator output. The adjustment of the input stimulus current, when required to achieve the criterion value, requires the application of a scaling factor, *S*, to transform changes of input current in dB to changes of excitation in dB.

Given psychophysical data showing the effects of the stimulus parameter under investigation, the Temporal Model can be used to infer the physiological effects of the parameter on neural excitation in step 1, and the scaling factor in step 4, that are needed to fit the predictions to the actual data. Thus, the Temporal Model potentially provides insights into how individual peripheral neural factors can influence temporal effects on loudness. Some examples of this process are described below.

**FIGURE 3:**
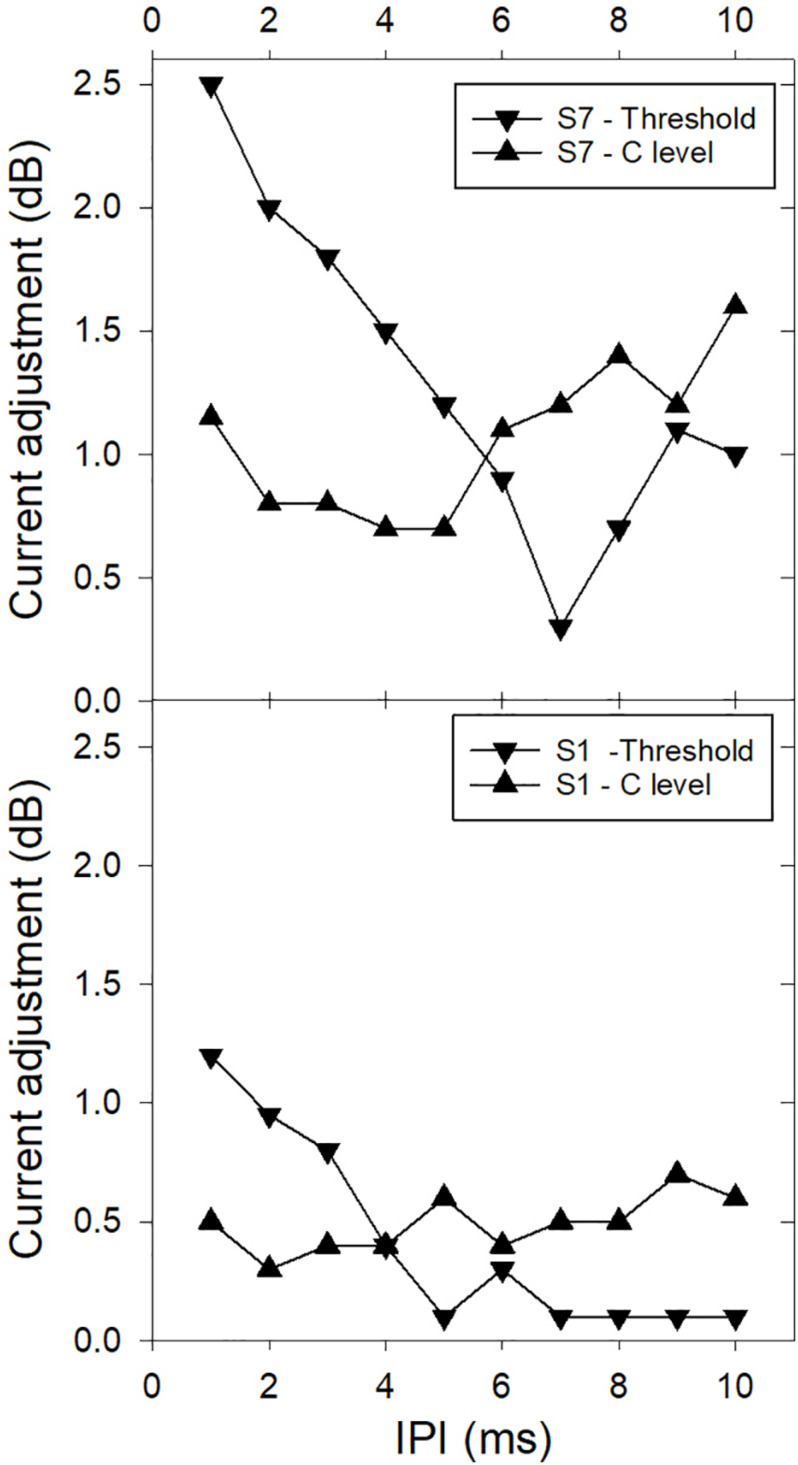
Examples from two CI users showing the effect of interpulse interval (IPI) on loudness summation. The vertical axis shows the current reduction (in dB) needed to make the 2-pulse-per-period stimulus the same loudness (or threshold precept) as the single-pulse-per-period stimulus. The period was 20 ms. The two examples illustrate the non-monotonic effects that are variable between subjects and loudness levels (threshold or comfortable level – C) (Data redrawn from McKay and McDermott, 1998).

McKay and McDermott (1998) applied the Temporal Model in three experiments that investigated the effect of IPI on detection and loudness. In these experiments, IPI was varied and equal-loudness or threshold functions were measured by adjusting the stimulus current. In experiments 1 and 2, a second pulse was inserted into each period of a 50 or 250 Hz pulse train, respectively, with a varying IPI between the two pulses in each stimulus period, and in experiment 3, constant-rate stimuli were varied in rate. Figure 3 shows representative results of experiment 1 for two CI users, illustrating both the non-monotonic effect of IPI on loudness and inter-listener differences. The non-monotonic effect of IPI on loudness is a result of the counteracting influences of refractoriness on the second of each pulse pair and the shape of the integration window. A smaller IPI reduces the excitation evoked by the second pulse, but also increases the weighting of the second pulse in the integration window. The Temporal Model was used to fit the predicted effect of IPIs for each individual in experiment 1 to the measured data by modeling the relative excitation evoked by the second pulse of each pulse pair compared to that evoked by the first (step 1 of the model). It was found that the differences in the shapes of the functions of current adjustment for equal loudness in experiment 1 (as seen in Figure 3) could be successfully modeled by fitting parameters relating to peripheral neural factors in step 1 (the average refractory recovery time, and the proportion of available neurons that fired on the first pulse), with the scaling factor in step 4 adjusting the vertical scale of the functions. The central decision criterion applied in step 3 for equal loudness or threshold was equal maximum output of the integrator. The fitted scaling factor, *S*, in step 4 ranged between 1 and 6 and was significantly larger at higher current levels. Individual scaling factors from experiment 1 were successfully re-used for application of the model to the data for experiments 2 and 3. On average across CI users, the values of the predicted individual neural factors were consistent with a large proportion of neurons being activated close to their individual thresholds for the current ranges used – with low spike probabilities (around 0.7) and long mean relative refractory times (average 5.5 ms). The variation of these factors between subjects can be hypothesized to be associated with neural survival density and the health of the surviving neurons.

In McKay et al. (2013b), the Temporal Model was further successfully applied to psychophysical data from CI users to understand the effects of modulation frequency on modulation detection (i.e., temporal resolution), the effect of stimulus duration on loudness, and the influence of masker-probe time interval on probe threshold in forward masking experiments. The decision criterion applied for the effect of modulation frequency on modulation detection was a fixed modulation depth of the integrator output for different modulation frequencies. For the effect of duration on loudness, the decision criterion was that the maximum integrator output for different durations was equal to that for the first pulse on its own. For the effect of masker-probe time interval on forward masked probe thresholds, the criterion was a fixed maximum difference between integrator outputs with and without the probe stimulus (which occurred near the probe offset). It is notable that all of the data across the different psychophysical experiments in CI users were successfully predicted by the model using the central integration window identical to that used to predict data in similar acoustic experiments, and with consistent model fitting parameter values across experiments. As in McKay and McDermott (1998), it was clear that the scaling factor of current to excitation (in dB/dB) needed to fit the experimental data increased for stimuli with higher absolute current levels (i.e., excitation was not a fixed power function of current over an extended range of currents). The increase in *S* at higher levels is likely to be due to the higher currents accessing more tightly packed but distant axonal processes compared to the sparse peripheral processes in the deaf cochlea, as also proposed by Nelson et al. (1996) based on intensity discrimination experiments. The fact that the normal-hearing central temporal integration window could be used without adjustment to explain the measured data implies that temporal resolution is essentially normal in CI users, as measured by the low-pass cut-off frequency of temporal modulation transfer functions, which is determined by the integration window shape.

In contrast, by applying the same phenomenological model to data from the same psychophysical experiments for users of the auditory mid-brain implant, McKay et al. (2013b) demonstrated that electrically stimulated neurons in the inferior colliculus must behave quite differently to peripheral auditory neurons (a higher average spike probability, close to 1, and shorter average recovery time of 1–2 ms) and that the normal-hearing central integration window needed to be considerably widened to explain the psychophysical data. Additionally, a large degree of adaptation had to be included in the first model step to explain the effects of masker duration on forward masking (an inclusion that was not necessary for CI users).

### Clinical Application of the Temporal Model: Objective Fitting of CIs

All modern implant designs enable the measurement of electrically evoked compound action potentials (ECAPs) – the whole-nerve response of the auditory nerve to individual current pulses – using implanted intracochlear electrodes as measurement electrodes. The use of ECAPs in automatic or objective programing of CIs has been limited by the very modest correlation between ECAP thresholds and the psychophysical data required for programing. The latter data are the current levels on individual electrodes required to attain hearing threshold and comfortably loud sensations for pulse trains at the sound processor stimulation rate (usually at least 500 Hz). Although hearing thresholds of single-pulse stimuli, or pulse trains with very low rate (e.g., 40 Hz), are highly correlated with ECAP thresholds (Brown et al., 1996), the correlation reduces as the rate of stimulation for the psychophysical measurement increases (Brown et al., 2000; Hughes et al., 2000; Cafarelli Dees et al., 2005). The decrease occurs because the slope of the threshold (or equal loudness) versus rate function (see Figure 2) varies across people in a way that cannot be predicted from the ECAP measurement for an isolated pulse. Therefore, ECAP thresholds for isolated pulses cannot be used on their own for totally objective programing. To achieve objective programing using ECAP thresholds, additional objective information about the shape of the behavioral threshold versus rate function is needed.

**FIGURE 4:**
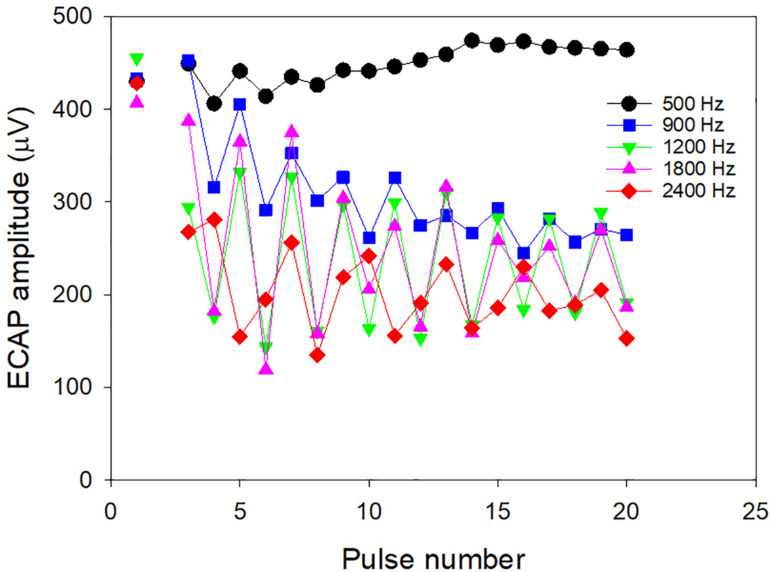
ECAP amplitudes to individual pulses in continuous pulse trains with different rates of stimulation. The unconnected symbols on the left are amplitudes to individual pulses as per the usual clinical measurements of ECAPs. The data was collected by McKay et al. (2013a).

The relation between the total excitation evoked by an isolated current pulse and the loudness evoked by a high-rate pulse train (the latter needed for CI programing) can be predicted for an individual by the Temporal Model if we know how the evoked excitation varies for each pulse in a high-rate pulse train for that individual. If we could objectively measure the latter (instead of modeling it in step 1) then the slope of the individual behavioral threshold versus rate function could be predicted by the Temporal Model. The slope, in turn, would allow the high-rate threshold to be estimated given the low-rate threshold predicted from the low-rate ECAP threshold. McKay et al. (2013a) used a high-rate subtraction technique (Hay-McCutcheon et al., 2005) that allows ECAP amplitudes to be measured for individual pulses within an ongoing high-rate pulse train. They hypothesized that the relative excitation evoked by each pulse in the pulse train (see example in Figure 4) is linearly correlated with the relative ECAP amplitudes evoked by the same pulses, and that therefore these subject-specific relative ECAP amplitudes can be inserted into step 1 of the Temporal Model to predict individual differences in the slope of the behavioral threshold versus rate functions. The results showed that, for rates above 500 pps, where refractory effects and temporal integration have the most influence on loudness, the average ECAP amplitude changes (averaged across subjects) predicted the average behavioral slope well, but neither varied significantly between participants. Instead, for rates below 500 pps, where very little reduction in excitation occurs after the first pulse (Figure 4), there was large variability between participants in the slope of the behavioral threshold versus rate function. The differences between subjects could be fitted by the Temporal Model by adjusting the scaling factor, S, between current and excitation to increase more steeply with level in individuals with a flatter threshold function below 500 Hz. Based on the idea that a steep increase in S may be associated with activation of more distant axonal processes, it was hypothesized that individuals with a flatter behavioral function below 500 Hz were those with poorer survival of peripheral processes (thus needing higher currents to achieve the same loudness compared to those with better neural survival). Indeed, animal studies have shown that the effect of rate on threshold for low rates is correlated with cochlear health (Pfingst et al., 2011).

Based on the results of McKay et al. (2013a) it was hypothesized that an objective measure of neural health might be combined with standard ECAP thresholds to improve the prediction of high rate behavioral thresholds for objective programing. McKay and Smale (2017) tested this hypothesis, by measuring the current offset (in dB) between ECAP amplitude growth functions evoked by stimulus pulses differing in phase duration or IPG duration. These objective measurements have been correlated with spiral ganglion cell survival in animal studies (Prado-Guitierrez et al., 2006; Ramekers et al., 2014). Brochier et al. (2020) have presented a theoretical model to explain the effects of IPG on ECAPs, and applied it to previous animal and human data. They argued that the ECAP function offset measurement (as used by McKay and Smale) is correlated with neural health (i.e., the health status of surviving neurons) as distinct from neural density (or number of surviving neurons), although these two aspects of cochlear health are likely to be correlated with each other, particularly in animal studies, due to the deafening techniques used. Consistent with their own hypothesis, McKay and Smale (2017) showed that the ECAP function offset (averaged across electrodes) was modestly correlated across subjects with the average slope of the behavioral thresholds versus rate function for rates between 40 and 1,000 Hz, but not the slopes for rates higher than 1,000 Hz. Thus, subjects with flatter low-rate function slopes on average across the electrode array were those with poorer health of surviving neurons, as measured by the ECAP offset.

With regard to the slopes of the ECAP amplitude growth functions, McKay and Smale (2017) found that, within individual subjects, electrodes with higher behavioral thresholds had greater ECAP slopes (expressed in μV/dB). This result is consistent with the observation of McKay et al. (2013b) that high current levels for high-rate stimuli are associated with a faster increase with level of the scaling factor *S* (excitation growth with current on a dB/dB scale). It is interesting to note that Brochier et al. (2020) argue that the ECAP amplitude growth function slope measured in dB/dB is not related to either neural survival density or health of the surviving neurons. The same would apply to the ECAP slopes in μV/dB measured in McKay and Smale (2017) since they were calculated over identical ranges of ECAP amplitudes for different stimulus conditions. Consistent with this observation, the ECAP offset measurement was not correlated with the ECAP amplitude growth function slope, and the ECAP slopes did not predict any across-subject variations in absolute behavioral thresholds or slopes of the threshold versus rate functions. Overall, use of both measures together improved the prediction of high-rate behavioral thresholds using ECAP measures alone. For example, for behavioral thresholds at rates of 1,000 Hz, the correlation between predicted and actual thresholds increased from *r* = 0.47 (*p* = 0.12) to *r* = 0.70 (*p* < 0.001) when the ECAP offset and ECAP slope were used as predictors in addition to the ECAP threshold.

## Multi-Electrode Stimuli

### Loudness of Multi-Electrode Stimuli and the Detailed Model

In normal CI use, multiple electrodes are activated in quick succession. It is therefore important to consider how loudness is summed across different places in the cochlea for interleaved electrical pulse trains. McKay et al. (2001) studied loudness summation for two interleaved pulse trains, measuring the influence on loudness summation of electrode separation, pulse repetition rate, and overall current level. In the experiment, two pulse trains on two different electrodes were first loudness balanced, and then interleaved. The current reduction (in dB) in the dual-electrode stimulus needed to equate its loudness to that of each component single-electrode stimulus was used as the (relative) measure of loudness summation. Surprisingly, the effect of electrode separation was very small, and, in addition, varied in direction, with some participants showing a reduction in loudness as the electrode separation was decreased and some showing an increase in loudness. Analogs to the effect of temporal separation described in section “Single-Electrode Stimuli,” the results were consistent with two counteracting effects of spatial electrode separation. A phenomenological model (labeled here as the “Detailed Model”) was proposed to explain the results of these experiments, in which the loudness of stationary (time invariant) electrical stimuli is determined by three steps as follows:

1. Using the Temporal Model, neural activity at each cochlear place is integrated using the sliding central temporal integration window. The output of this step is a spatial “excitation density” function that can vary over time, but will be relatively constant for a stationary stimulus.
2. The excitation density function from step 1 is transformed to an instantaneous “specific loudness” function (i.e., loudness arising from each place in the cochlea at that instant). The function that performs this transform relates neural activity to loudness.
3. The specific loudness is then integrated across cochlear place, similarly to the integration of specific loudness in acoustic models of loudness (Moore and Glasberg, 1997), the result of which is the overall loudness of the stimulus.

When electrodes are in close proximity, the overlap of the neural populations stimulated by each electrode is increased, leading to reduced overall neural activation in step 1 due to neural refractoriness. If loudness were linearly related to the total amount of evoked neural activity (i.e., the transform in step 2 was linear), then loudness would always decrease as electrode separation is decreased. The finding that loudness does not systematically decrease, however, leads to the conclusion that the transform in step 2 is non-linear and expansive (e.g., a power or exponential function). In that case, excitation density functions that are more localized (same total excitation but over a smaller area) would produce a greater loudness than ones that are more spatially spread. Thus, if neural refractoriness was not present in step 1, loudness would always systematically increase as electrode separation decreased. The two effects together lead to no, or little, effect of separation on loudness, as seen in the psychophysical data.

The application of the Detailed Model requires knowledge of individual characteristics of the spread of activation and the response properties of the activated neurons, both of which are likely to vary considerably between different people and places in the cochlea. However, these properties can be inferred from physiological data or psychophysical data, as described in section “Single-Electrode Stimuli,” to apply the model in different conditions to explain how loudness varies for different stimuli. A second, practical, way of applying the model without the need to find the details needed in step 1 can be derived from the fact that there was very little effect of electrode separation on loudness in McKay et al. (2001). This method of applying the Detailed Model, which we will designate the “Practical Method” (McKay et al., 2003), is described below.

#### The Practical Method for Predicting the Relative Loudness of Electrical Stimuli

The development of the Practical Method used the approximation that there is no effect of electrode separation on loudness, together with the assumption that individual current pulses of a complex stimulus that do not produce spatially overlapping effects in the cochlea contribute independently to the overall loudness. The latter assumption is based on acoustic models of loudness (Zwicker and Scharf, 1965; Moore and Glasberg, 1997) in which loudness contributions from non-overlapping cochlear filters contribute additively to the total loudness. Since the loudness-addition step of acoustic models refers to loudness processing at stages more central than the cochlea, it is reasonable to presume that the same central process applies in electrical hearing. If pulses evoking non-overlapping neural excitation patterns contribute independently to loudness, and the overall loudness does not change with the degree of overlap, then electrical pulse trains must always behave *as if* the loudness contributions from different current pulses are independent, regardless of whether they are widely or closely spaced in the cochlea. In other words, if the effect of overlapping neural activation patterns on loudness is not significant, and can be approximated as zero, then the loudness evoked by the different pulses must always add similarly to the case when the activation patterns do not overlap, and the pulses must contribute additively and independently to the overall loudness, no matter where they occur on the electrode array.

**FIGURE 5:**
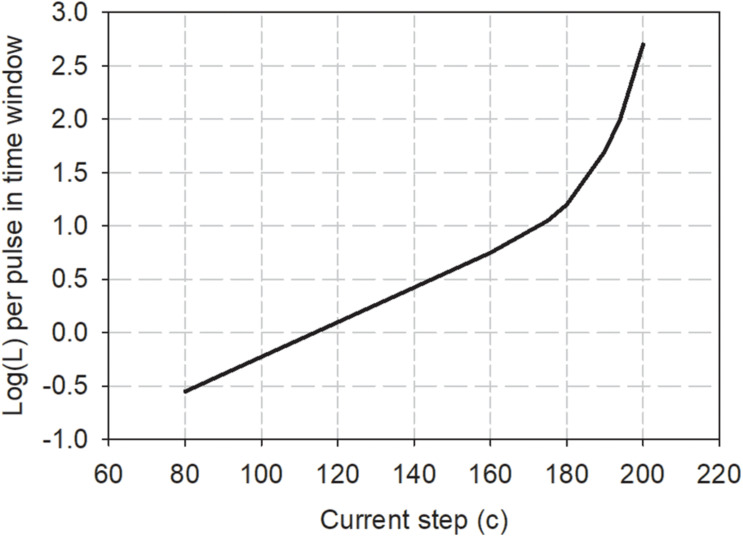
Loudness growth function for one CI user derived from loudness summation experiments as described in the text. To use the Practical Method to estimate the loudness of any electrical pulse train, the loudness contribution of each pulse in 2 ms windows is obtained from the graph and summed to estimate the loudness of the stimulus relative to a reference stimulus of loudness 100 (Data redrawn from McKay et al., 2003).

The Practical Method proposes that a running estimate of loudness (defined here as “instantaneous loudness”) relative to the loudness of a reference stimulus can be obtained by summing the loudness contributions of each pulse in small reference time windows (e.g., a 2 ms rectangular window). The loudness contribution of each pulse (*L*) is calculated from a loudness growth function of log(*L*) versus clinical current level (*c*). The loudness growth function for each electrode can be determined experimentally using the assumption that a stimulus that has two equal-current pulses in one period has twice the loudness of a stimulus with one pulse per period. The slope of the loudness growth function at that particular current level is then determined by the current adjustment needed to loudness balance the two stimuli. By measuring the slope at multiple absolute current levels and using different rates of stimulation, a complete growth function can be derived. An example of such a loudness growth function is shown in Figure 5, and is characterized by Eq. 2:

(2) $$L\,o\,g\,\left(L\right)=a\times c+\left[0.03\times b\times e^{\frac{\left(c-c_{0}\right)}{b}}\right]+k,$$

where *a*, *b*, and *c*_0_ are fitting parameters. The parameter *a* is the slope of the linear portion of the function and applies when *c* is less than *c*_0_, the latter defining the knee-point above which the function becomes expansive. The arbitrary constant, *k*, can be used to set the loudness of a reference stimulus to an arbitrary loudness value. In the experiment to derive Eq. 2 (McKay et al., 2003), clinical current levels were used, which are equal to logarithmic steps of 0.176 dB for the CI24M implant used. The relation between current level (*c*) and current (*i*) in μA is given by the formula (provided by the manufacturer):

(3) $$i=10\times 175^{c/255}$$

It can be seen that the relation between loudness, *L*, and current, *i*, can be described as a power function (with exponent *a*) for low currents (*c* << *c*_0_), when the second term in the equation becomes essentially zero. Low currents will usually apply when the rate of stimulation is high, for example, at the output of most clinically used sound processors. McKay et al. (2003) found that the slope *a* did not vary very much between participants. When the current is expressed in dB instead of clinical current units, the linear slope, *a*, had a mean of 0.1 *log(L)* per dB; in other words, loudness increased by a ratio of 1.26 for every dB increase in current in the linear part of the loudness growth function. This value of *a* can also be estimated from the slope of threshold versus rate functions for rates above 900 Hz (where absolute current values are low). For example, analysis of average high-rate slopes in the threshold data in Figure 2 of McKay et al. (2013a) produces the same value of *a* = 0.1, when current is expressed in dB.

An extended simplification is possible when predicting the relative loudness of high-rate stimuli, where the first term can be used on its own with *a* = 0.10 if expressing current in dB, without the need to generate participant-specific values of the other fitting parameters. The exponential term, which only becomes significant at higher current levels, is likely related to the increase at higher current levels of the scaling factor (*S*) described above that is needed to fit psychophysical data using the Temporal and Detailed Models. If we assume that loudness is a power function of neural excitation, as is common when relating psychophysical percepts to physiological data, then it can be inferred from Eq. 2 that the transform from current to excitation is also a power function for low currents (i.e., a constant exponent, *S*), but that for currents past the kneepoint, *c*_0_, *S* will increase with increasing absolute current.

In McKay et al. (2003), two psychophysical experiments were carried out to validate the Practical Method using multi-electrode periodic stimuli with a period of 2 ms (which can be considered perceptually time invariant). In the first experiment, dual electrode 2-pulse-per-period stimuli were created in which the relative currents of the two pulses were varied and the stimuli loudness balanced against the reference stimulus, which comprised equally loud pulses on the two electrodes. The predicted loudness (derived from the Practical Method) of the balanced stimuli relative to the reference stimulus was constant, as expected, as the relative currents were varied. In the second experiment, 54 arbitrary stimuli of differing overall loudness were created, which had from 1 to 8 pulses in the 2 ms period, and where each pulse could be on an arbitrary electrode with arbitrary current value (within the dynamic range of the participant). A reference stimulus on a central electrode was balanced against each of the 54 stimuli and the balanced current of the reference was compared to that predicted by the Practical Method. The average difference between predicted and actual balanced current of the reference stimulus was very small, being only 0.2 clinical current levels (0.035 dB).

A third validation experiment was carried out by McKay and Henshall (2010), who investigated the effect of amplitude modulation on the loudness of single-electrode stimuli. In that experiment, modulated stimuli had different carrier rates (0.5, 1 or 8 kHz), different modulation rates (500 or 250 Hz), different modulation depths, and different overall levels (threshold, 60 and 90% of the dynamic range). The Practical Method was used to predict the effects of carrier rate, modulation frequency, and overall level on the current of the unmodulated stimulus of the same carrier rate that was equal in loudness to the modulated stimulus. The model correctly predicted that, for stimuli with low currents (the 8 kHz carrier rate stimuli at all levels in the dynamic range, and the threshold stimuli with lower carrier rates), the equally loud unmodulated stimulus had a current equal to the average current in the modulated stimulus. This finding is consistent with these stimuli having low enough currents to fall onto the linear part (in log/log coordinates) of the loudness growth function (Eq. 2 and Figure 5). The other stimuli (500- and 1,000-Hz carrier rates at 60 or 90% DR) comprised pulses with higher currents that fell into the non-linear expansive part of the loudness growth function, and both model and psychophysical data showed that the current of the equally loud non-modulated stimulus was greater than the average current of the modulated stimulus and moved closer to the peak modulated current as the absolute level of the stimulus increased (carrier rate decreasing or level in the dynamic range increasing). The insights provided by this study showed that it was important, when determining modulation detection ability in CI users, to take into account systematic differences in loudness between modulated and unmodulated stimuli, as loudness differences will provide confounding cues to the presence of modulation, leading to overestimation of modulation detection abilities.

This overestimation of modulation detection ability was demonstrated by Fraser and McKay (2012), who measured a series of temporal modulation transfer functions (modulation detection threshold versus modulation frequency) while limiting the use of loudness cues. In the experiment, the target (modulated) stimulus was loudness balanced with the standard (unmodulated) stimulus, and level jitter was used to additionally limit use of loudness cues. Previously studies investigating modulation detection in CI users had set the current in the reference unmodulated stimulus to the average current in the modulated stimulus. The loudness cues in the latter case would become more salient as the modulation frequency is increased, when larger modulation depths are needed. Thus, loudness cues led to overestimation of modulation detection ability, particularly for high-frequency modulations, thus underestimating the low-pass characteristics of the modulation transfer functions. The functions measured by Fraser and McKay (2012) had low-pass cut-off frequencies broadly consistent with those for normal hearing subjects. The facts that low-frequency cut-off frequencies are broadly in the normal range, and that the central temporal integration window used in the Temporal Model is the same as for normal hearing, suggest that temporal resolution is largely unaffected in CI users. These results suggest that the differences between CI users in absolute measures of modulation detection ability at low modulation frequencies, which have been related to differences in speech perception ability (Luo et al., 2008; Arora et al., 2011; Won et al., 2011; Brochier et al., 2017), are related more to variance across subjects in intensity difference limens (McKay et al., 2018) or modulation sensitivity than to variance in temporal resolution.

#### Extensions of the Practical Method

The Practical Method as derived by McKay et al. (2003) is able to output a running estimate of loudness in small increments of time by summing loudness contribution from each pulse. For a perceptually stationary stimulus, this estimate will suffice to deduce the overall loudness of the stimulus (relative to that of a reference stimulus). However, if the stimulus is dynamically changing over time, a further question would be how to derive the overall loudness perceived from the time-varying estimates output by the Practical Method. This question has been addressed in a study by Francart et al. (2014), who investigated how existing acoustic models for predicting the loudness of time-varying signals can be adapted to extend the Practical Method to predict the overall loudness of time-varying electrical signals in CIs. Two methods were described that well predicted the psychophysical data, both of which first calculated the “instantaneous loudness” by integrating the individual pulse loudness contributions (as defined by the Practical Method) over a sliding temporal integration window. In both cases, the shape of the integration window was defined as in Eq. 1, and the Equivalent Rectangular Duration (ERD) of the window was used as a fitting parameter. The first method investigated by Francart et al. (2014) that fitted the experimental data well used an integration window with ERD of 2 ms and then calculated long-term loudness from the varying instantaneous loudness following the method of Glasberg and Moore (2002), which entailed application of an automatic gain control like circuit to the instantaneous loudness values, with an attack time of 22 ms and a release time of 50 ms to obtain short-term loudness values, followed by application of a second automatic gain control like circuit to obtain long-term loudness values. The second successful method described by Francart et al. (2014) was simpler than the first, and used a temporal integration window with ERD of 4.3 ms to obtain the “instantaneous loudness” and then defined the 99th percentile of instantaneous loudness as the long-term loudness. Note that these integration windows have a smaller ERD than that used in the Detailed Method. These ERDs are not inconsistent with the Detailed Method, since the latter integrates peripheral neural activity, while the practical methods integrate loudness contributions. Since the transform between neural activity and specific loudness in the Detailed Method is non-linear and expansive, it would be expected that the ERD that best fits loudness integration data would be smaller than that which fits neural activity integration data.

The Practical Method also cannot be directly applied to pulsatile stimuli in which the pulses occur simultaneously rather than sequentially, for example, in certain signal processing strategies or in simultaneous analog stimuli. For these stimuli, an additional effect must be included when predicting loudness: the direct summation of simultaneous currents at the neural interface (Shannon, 1983; Tang et al., 2011). This effect is highly dependent on the distance between electrodes and the spatial spread of currents in individual cochleae. For example, Marozeau et al. (2015) compared simultaneous with sequential stimuli using monopolar and focused multipolar modes of stimulation. They found that stimuli in the multipolar mode, which is designed to produce a highly focused current field, produced only small differences in loudness between simultaneous and sequential conditions, whereas the monopolar stimuli needed current adjustments of up to 4 dB to make the simultaneous and sequential stimuli the same loudness.

In the case of stimuli with simultaneous biphasic pulses, the Practical Method could still be used if psychophysical loudness summation data due to current summation for the stimulus conditions used (e.g., mode of stimulation and electrode distance) were obtained and included in the model. An example of such an adaptation of the Practical Method was demonstrated by Langner et al. (2020), who measured loudness summation caused by current interaction of simultaneously activated pairs of virtual channels. Virtual channels simultaneously activate two adjacent intracochlear electrodes to steer the peak of the current field to positions between the physical electrodes. Paired virtual channels therefore activate four intracochlear electrodes simultaneously. Such paired virtual channels are used in the “Optima-Paired” sound coding strategy of Advanced Bionics. In the adaption of the Practical Method, Langner et al. (2020) balanced the loudness of paired-channel stimuli to those of the component single virtual channels to create a model of how channel distance, and relative currents in the component channels of each pair, influence the loudness. This additional model was then incorporated into the Practical Method to predict the loudness of paired-channel stimulation strategies compared to strategies that sequentially activated virtual channels. To do this prediction, the loudness contribution of each paired-channel pulse pair was replaced in the Practical Method calculation of the loudness by an equivalently loud single-channel pulse with current determined by the model derived from loudness balancing data. This method of Langner et al. (2020) provides a way for clinicians to automatically adjust the program of the sound processors when switching between paired-pulse and fully sequential signal processing strategies. To do this adjustment when changing to the paired strategy from a sequential strategy, clinicians can lower the current range assigned to each virtual electrode (which is determined for sequential stimulation using each virtual channel separately) by an amount predicted by the model calculation, so that the simultaneous stimulation does not produce sounds that are too loud.

### Clinical Applications of the Loudness Models to Signal Processing Strategies

The Practical Method of loudness estimation has been applied to several novel signal processing strategies that aim to create more control of overall loudness and frequency-specific contributions to loudness (specific loudness). Current clinically used processing strategies assign a fixed electrical dynamic range to each electrode, based on single-electrode psychophysical measures of loudness. However, this technique does not take into account the loudness summation that occurs when multiple electrodes are activated concurrently in normal implant use, leading to sounds of different bandwidth or overall levels producing loudness percepts that vary in ways that are quite different to what an acoustic listener would hear.

**FIGURE 6:**
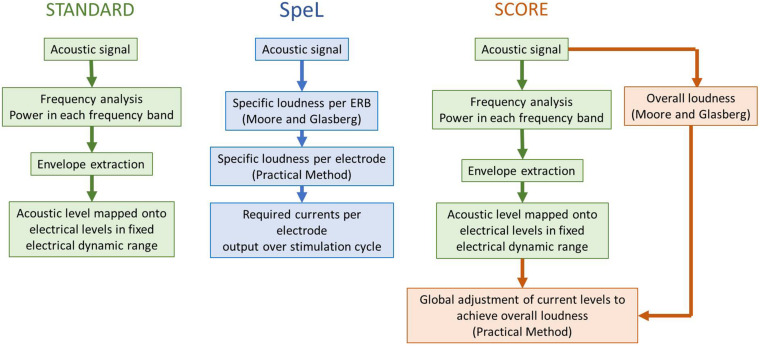
Schematic that illustrates the essential principles of the signal processing strategies SpeL and SCORE in relation to standard clinically used signal processing strategies. The flowchart illustrates the differences in processes that occur for each stimulation cycle or within each update window. The reader is referred to the original articles (McDermott et al., 2003; Varsavsky and McDermott, 2013) for a detailed technical description of the strategies.

The first signal processing strategy to use the Practical Method to control loudness was the SpeL strategy (McDermott et al., 2003), which utilized the acoustic loudness model of Moore and Glasberg (1996, 1997) to convert the incoming signal into specific loudness in each cochlear equivalent rectangular bandwidth (ERB), following which the specific loudness was converted using the Practical Method to the required current values on electrodes across the array (Figure 6). Cochlear ERBs divide the cochlea into non-overlapping sections with characteristic frequency ranges related to the width of cochlear filters at the same frequencies, and each electrode was assigned a constant 1.3 contiguous ERBs. Thus, in the SpeL strategy, the specific loudness pattern of the incoming acoustic signal (calculated for a person with normal hearing) was replicated as the specific loudness pattern produced by electrical pulses across the electrode array, effectively “normalizing” the relative overall loudness of incoming sounds, and the relative loudness contributions of different frequencies within the sound. McDermott et al. (2003) implemented SpeL in a wearable research processor and used a loudness estimation psychophysical task for participants wearing the research processor to compare the predicted and estimated loudness of acoustic noise bands of various bandwidths and levels. The results confirmed that SpeL restored the relative loudness of different bandwidths and different intensities to that experienced by normal-hearing listeners. McDermott et al. (2005) showed that, after 4 weeks trial use of SpeL, CI users had equivalent speech understanding in quiet and noise to their clinical strategy (ACE), while improving the audibility of soft sounds by an average of 5 dB. In the ACE strategy, soft speech will activate fewer electrodes than louder speech, as frequency bands with very low levels produce no stimulation. This drop in number of activated electrodes leads to an uncompensated reduction in loudness summation across electrodes, causing the soft speech to be too difficult to hear. In contrast, the SpeL strategy calculates the correct (or “normal”) overall loudness of the speech and automatically adjusts the currents to produce the correct loudness.

The SpeL strategy required individual loudness growth functions (like Figure 5) to be measured on each electrode and also required the frequency-to-electrode allocation to be altered away from that which the participants were used to in the ACE strategy, so that each electrode received information from an a constant 1.3 contiguous ERBS. Although the total range of assigned frequencies across the electrode array were as closely matched as possible to the participant’s usual range of assigned frequencies, there remained a significant shift in assignment toward the middle of the array. Thus, failure to adapt fully to the change of electrode assignment may have influenced the results of the speech test in McDermott et al. (2005). These considerations led to the development of a second strategy based on the Practical Method – SCORE (Varsavsky and McDermott, 2013). Instead of replicating the acoustic *specific* loudness pattern in the electrical stimulation across electrodes, SCORE aimed to only control the instantaneous *overall* loudness (Figure 6). It did this by estimating the incoming instantaneous loudness using the acoustic models of Moore and Glasberg (1996, 1997), and adjusting the output current levels (equally across electrodes) of the ACE strategy to match the acoustic instantaneous overall loudness, using the Practical Method. Since SCORE only acts upon the output of a signal processing strategy, it can be combined with any signal processing strategy (not solely ACE, as used by Varsavsky and McDermott, 2013) to control overall loudness. It can therefore take advantage of features of processing strategies (such as the noise reduction benefit of maxima selection in ACE) while normalizing overall loudness percepts. Varsavsky and McDermott (2013) implemented SCORE for experienced users of the ACE strategy and demonstrated that soft speech (50 dB SPL) was more intelligible with SCORE than with the ACE strategy (a mean increase of 8.8 percentage points). Since SCORE matches instantaneous acoustic loudness with instantaneous electric loudness, it has an ideal application in bimodal hearing, in which CI users use a hearing aid on the non-implanted ear. SCORE-Bimodal was developed and tested by Francart and McDermott (2012b). It has the same SCORE processing as described above for the CI side, so that the instantaneous loudness (measured in time frames of 6.9 ms) of the electrical signal matches the instantaneous loudness of the acoustic signal at the CI microphone as predicted for normal hearing by the model of Moore and Glasberg (1997). On the hearing aid side, the predicted difference in loudness for normal hearing and hearing impaired listeners is computed by the model of Moore and Glasberg (1997) and used to adjust the gain of the hearing aid to match the normal-hearing loudness. Clinical assessment of SCORE-Bimodal (Francart and McDermott, 2012a) showed that it improved localization ability while maintaining speech perception ability in quiet and noise.

The Temporal and Detailed Models use the output of the sliding temporal integration window (integrated excitation) to predict perceptual decisions about modulation detection. Based on the model, modulation of rate of stimulation would lead to similar modulation of the integrator output as modulation of current amplitude. Brochier et al. (2018a) compared rate modulation detection with amplitude modulation detection and investigated the effects of modulation frequency and presentation level. They found that the two types of modulation detection were affected similarly by level and modulation frequency and were correlated with each other across the subject group. Following this result, Brochier et al. (2018b) devised a novel sound coding strategy (ARTmod) that coded amplitude modulations of the acoustic signal onto simultaneous rate and amplitude modulation in the electrical signal. They hypothesized that the two types of modulation would independently contribute to perception of amplitude modulations in acoustic speech signals, and thus it would be possible to use the added rate modulation to improve speech understanding. They found that speech perception improved with increasing amounts of rate modulation, which is consistent with rate and amplitude modulation being processed similarly and additively to transmit the acoustic amplitude modulation in the speech signal.

Finally, an adaptation of the Temporal Model was used by Lamping et al. (2020) to devise a signal processing strategy (designated TIPS) that removed pulses that were more likely to be masked by preceding pulses. The authors used the sliding integration window of Eq. 1 and applied it directly to the currents of the pulses in a continuous interleaved sampling (CIS) strategy, followed by a decision criterion that compared the integrator output with and without the pulse at the center of each window to decide whether to omit that pulse. Criteria of less than 1, 1.3, and 1.8 dB difference in integrator output were used to remove 25, 50, and 75% of current pulses, respectively. It should be noted that, since excitation is a power function of current (the scaling factor, S, in the model), applying the integrator to the current should lead to less variation in the integrator output than applying it to the excitation: therefore the criteria differences in output would be larger than those used in the study if the Temporal Model was used, and closer to the 3 dB criterion for detection used in acoustic studies of forward masking (Plack et al., 2002). However, since the criteria were used as an experimental variable, this difference does not have relevance to the results, which showed that the TIPS strategy improved speech perception in noise by 2.4 dB signal-to-noise ratio when removing 50% of the masked pulses.

## Conclusion

The application of phenomenological loudness models to psychophysical data of CI users has led to improved understanding of the influence of individual peripheral neural response behavior and neural health status on the transmission of features of the acoustic signal to the perception of the CI user. The knowledge gained has led to better understanding of differences in outcomes between CI users, and novel ways of determining cochlear health in CI users. The models have been applied to the development of novel signal processing strategies that aim to provide CI users with a more natural perception of loudness and better localization ability and to a novel way to improve the transmission of important amplitude modulations in speech to the CI listener.

## Author Contributions

CM is the sole contributor to this review.

## Conflict of Interest

The author declares that the research was conducted in the absence of any commercial or financial relationships that could be construed as a potential conflict of interest.
